# From Pulmonary Hyperlucency to Pulmonary Hypertension: A Case Report of Swyer-James-MacLeod Syndrome

**DOI:** 10.7759/cureus.87392

**Published:** 2025-07-06

**Authors:** Juan Pablo Montoya L, Juan Vasquez, Ricardo Uribe González, Manuela Guzman-Medina, Valentina Palacio-Arango

**Affiliations:** 1 Cardiology, Clínica Especializada EMMSA, Medellín, COL; 2 Radiology, Clínica Somer, Clínica Especializada EMMSA, Medellin, COL; 3 Radiology, Hospital Pablo Tobón Uribe, Clínica Especializada EMMSA, Medellín, COL; 4 General Medicine, Clínica Especializada EMMSA, Medellín, COL

**Keywords:** bronchiolitis obliterans, case report, pulmonary hypertension, swyer-james-macleod, unilateral pulmonary hyperlucency

## Abstract

Swyer-James-MacLeod syndrome (SJMS) is a rare condition characterized by unilateral pulmonary hyperlucency and vascular alterations secondary to post-infectious bronchiolitis obliterans. It is also an underdiagnosed disease due to the nonspecificity of its symptoms, which highlights the importance of complementary imaging studies for its identification. Although treatment is mostly conservative, in selected cases, surgical management can improve the prognosis and quality of life of patients. We present the case of an adult female with recurrent respiratory infections in childhood who had an incidental diagnosis of SJMS with pulmonary hypertension as a complication.

## Introduction

Swyer-James-MacLeod syndrome (SJMS), also known as unilateral hyperlucent lung, is a rare disease characterized by radiological hyperlucency, pulmonary hypoperfusion, and air trapping, usually secondary to post-infectious bronchiolitis obliterans in childhood [1,2]. It was first described in 1953 by Swyer and James, and later expanded by MacLeod in 1954 [1,2]. This entity has historically been underdiagnosed due to the nonspecificity of its clinical manifestations and variability in its presentation. A prevalence of up to 0.01% has been reported in radiographic studies, and its incidental finding is common in asymptomatic adults [3]. Physical examination is often nonspecific, and hence imaging studies are essential for diagnosis [3,4]. We present the case of an adult female with a history of recurrent respiratory infections in childhood, in whom an incidental diagnosis of SJMS was made, with pulmonary hypertension as an associated complication.

## Case presentation

A 70-year-old female patient was admitted to the hospital due to repeated syncopal events; she had been attending cardiology follow-up visits for suspected microvascular coronary artery disease (typical angina with a positive myocardial ischemia induction test and coronary angiography without evidence of obstructive lesions in the first three divisions of the coronary tree) and was carrier of bicameral pacemaker for sinus dysfunction. She also had a history of arterial hypertension, dyslipidemia, diabetes mellitus, and chronic obstructive pulmonary disease (COPD). She reported experiencing recurrent respiratory infections throughout her childhood, often requiring antibiotic therapy. She was admitted with vital signs within normal ranges, except for oscillating saturation between 88-90% on room air, with heart sounds with a fixed S2 and low-intensity vesicular murmur.

Previous studies were reviewed, including chest X-ray with eversion of the pulmonary artery cone, suggestive of pulmonary hypertension, in addition to transthoracic echocardiogram with severe eccentric ventricular hypertrophy with preserved left ventricular ejection fraction (LVEF) of 66%, increased end-fill pressure (averaged E/e': 13), severely dilated and hypertrophic right ventricle with an estimated tricuspid annular plane systolic excursion (TAPSE) of 24 mm, with severely dilated right atrium, minimal physiological mitral regurgitation, trivalve aortic valve with mild regurgitation, structurally normal tricuspid valve with mild regurgitation [peak velocity 3.77 m/sec - high probability of pulmonary hypertension, with echocardiographic Pulmonary to Left Atrial Ratio (ePLAR) of 0.3 m/s suggestive of precapillary pulmonary hypertension), pulmonary valve with mild regurgitation, and mean pulmonary pressure of 24 mmHg, with a dilated pulmonary artery of 30 mm.

**Figure 1:**
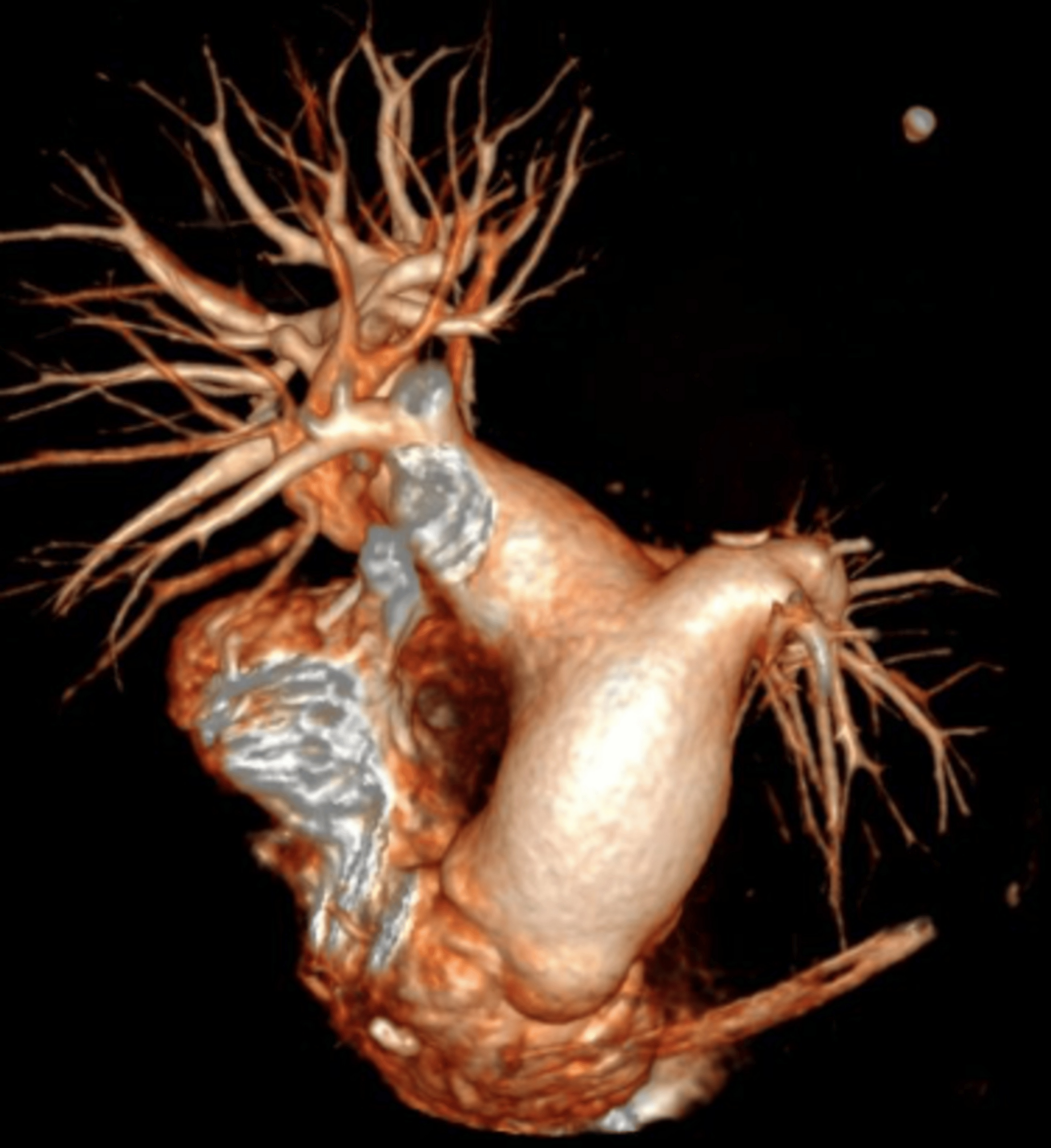
Angiotomography of pulmonary arteries with 3D reconstruction Prominence of the pulmonary artery trunk with hypoplasia, associated with a decrease in the number of distal vessels and thickness of the peripheral vasculature of the left lung

**Figure 2:**
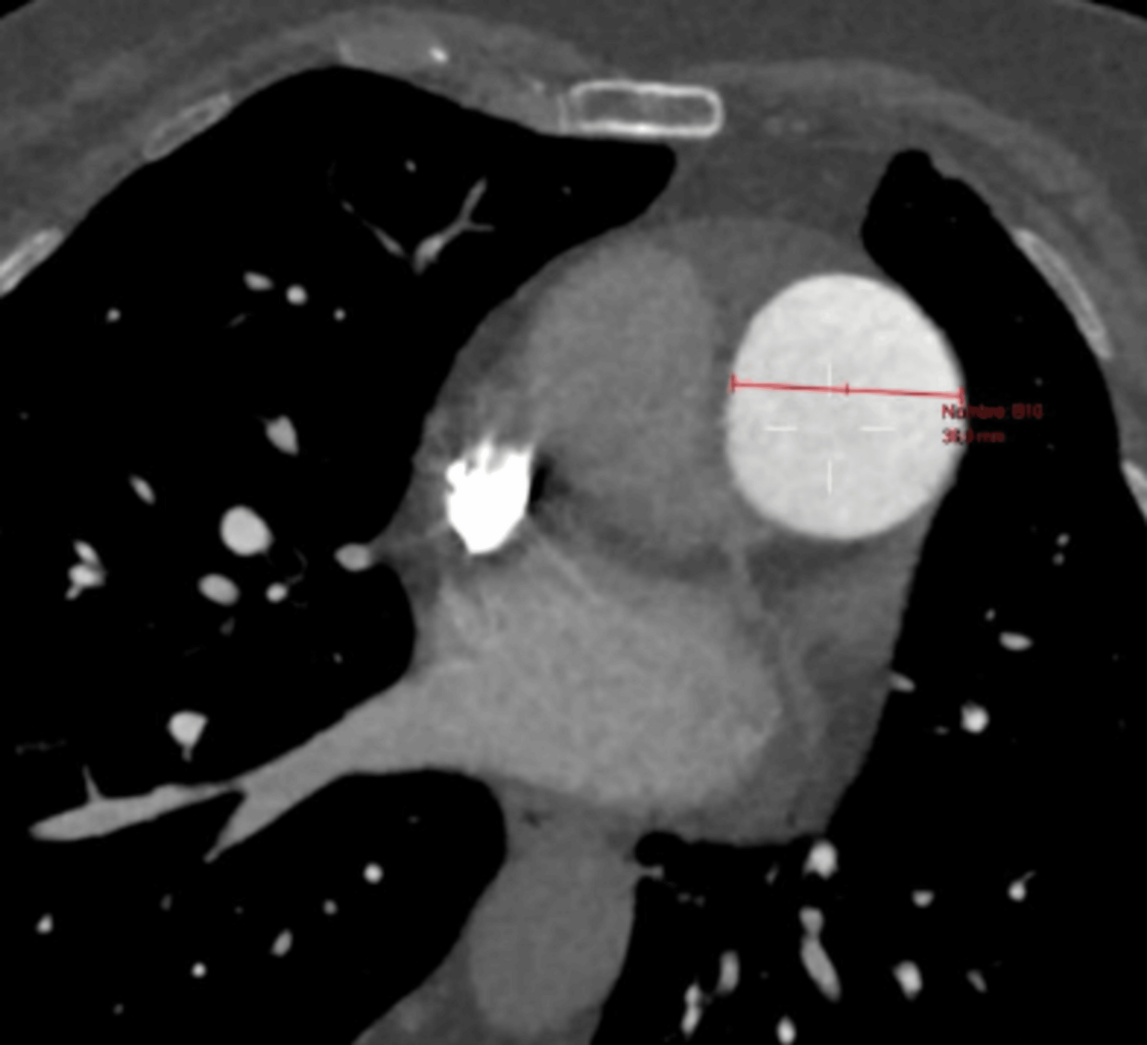
Angiotomography of pulmonary arteries - image 1 Soft tissue window with multiplanar reconstruction in pulmonary artery axis with increased pulmonary artery diameter as the only sign of right heart cavity pressure overload

**Figure 3:**
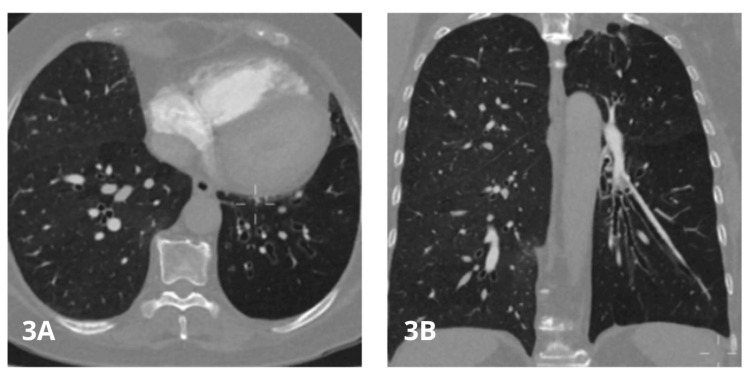
Angiotomography of pulmonary arteries - image 2 Window for pulmonary parenchyma in axial (3A) and coronal (3B) views; cylindrical bronchiectasis projected towards the left lower lobe

**Figure 4:**
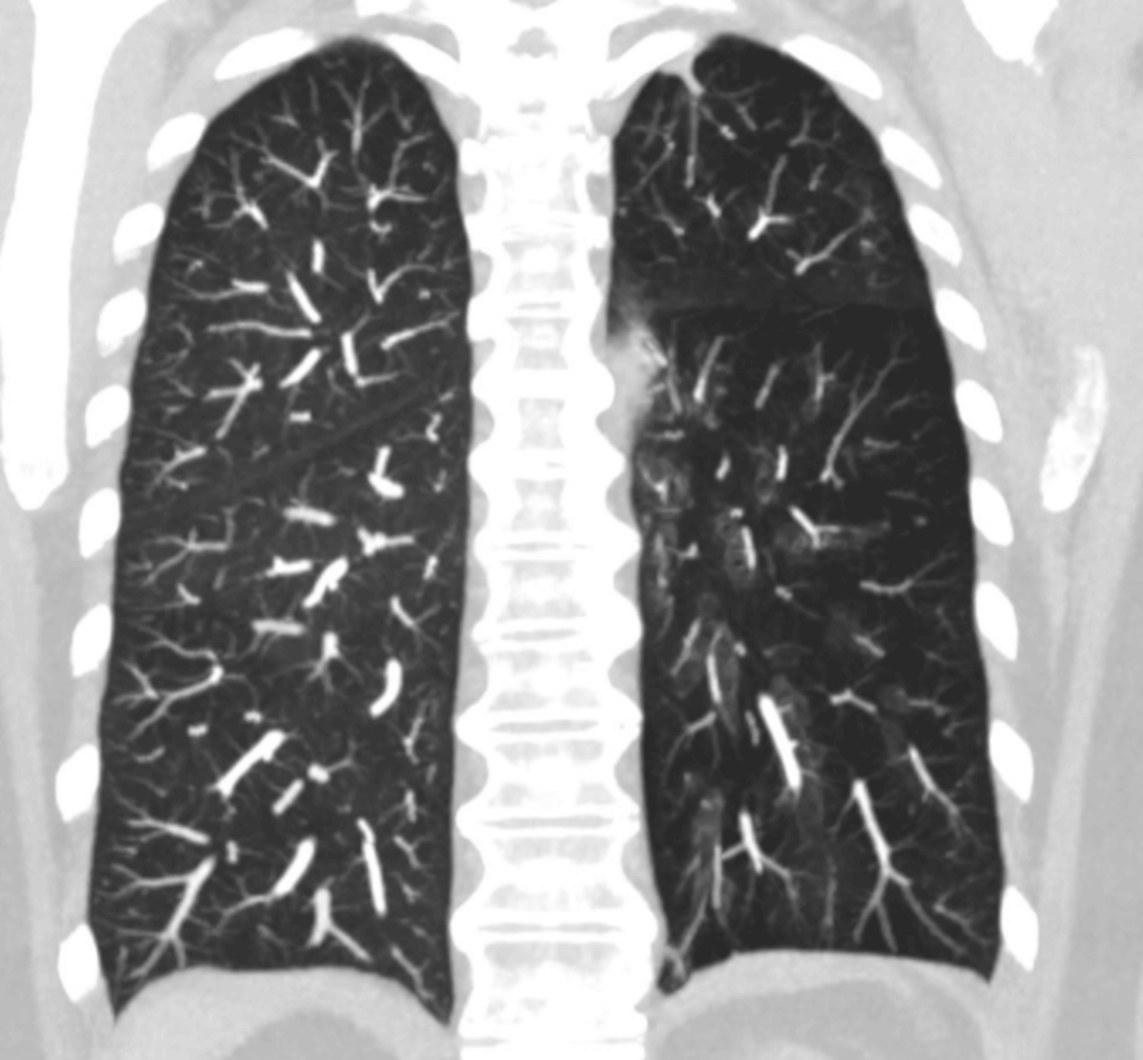
Angiotomography of pulmonary arteries - image 3 Coronal view of the pulmonary parenchyma with maximum intensity projections showing diffuse hypodensity of the entire left pulmonary parenchyma due to decreased caliber of the distal vessels

Given the absence of these findings in previous echocardiograms, the decision was made to perform chest angiotomography to look for acute or chronic pulmonary thromboembolism, which was ruled out; however, the procedure incidentally showed prominence of the trunk of the pulmonary artery as the only sign of pressure overload of right heart cavities, in addition to hypoplasia of the left pulmonary artery and a marked decrease in the number of distal vessels (Figures 1-2); all of these findings are associated with cylindrical bronchiectasis projected towards the left lower lobe (Figure 3-4), which pointed to SJMS.

The decision was made to perform the right catheterization. During the study, oximetric samples were taken, showing systemic output by the Fick method (Qs): 8.9 lt/mt; systemic vascular resistance (SVR) was calculated: 799 dyn (9.9 U Wood), and pulmonary vascular resistance (PVR): 323 dyn (4 U Wood); PAP: 80/28/46; PCP: 10 mmHg. These findings confirmed the diagnosis of moderate pulmonary hypertension. The patient was discharged with pharmacological management for pulmonary hypertension (ambrisentan 10 mg daily and sildenafil 25 mg every 12 hours). At the follow-up review one month later, there was improvement in functional class and long-standing respiratory symptoms previously reported by the patient.

## Discussion

In 1953, Canadian pediatrician Paul Robert Swyer and radiologist George James first described the initial findings of what is now known as Swyer-James-MacLeod syndrome (SJMS); the index patient was a six-year-old male with a history of recurrent respiratory infections since the age of three years. Imaging studies revealed hyperlucency and decreased vascular structure in the right lung field on chest X-ray, with normal bronchoscopy ruling out bronchial obstruction; angiocardiography revealed a normal heart, but with a smaller right pulmonary artery and decreased vascular filling to the ipsilateral lung [1]. A year later, in 1954, English pulmonologist William MacLeod provided more precise details by publishing a case series of nine adults with unilateral pulmonary hyperlucency [2], suggesting that its origin, although uncertain at that time, could be unearthed.

SJMS, also known as unilateral hyperlucent lung syndrome, is a rare condition with a reported prevalence of 0.01%, based on a study of 17.450 chest X-rays [3]. It is associated with episodes of post-infectious childhood bronchiolitis obliterans, caused by microorganisms such as Bordetella pertussis, Mycobacterium tuberculosis, Mycoplasma pneumoniae, Influenza A, Adenovirus type 3/7/21, Streptococcus pneumoniae, and Paramyxovirus mobillivirus [3-5]. These infections damage the bronchial epithelium, causing submucosal fibrosis with luminal obstruction, hypoplasia, and hyperinflation, with panacinar emphysematous changes of the bronchus compromised at the distal level. In addition, the loss of ciliary motility increases the risk of recurrent respiratory infections [6,7]. Its distinctive feature is hypoplasia and/or agenesis of the pulmonary artery [8], which causes hypoperfusion of the pulmonary parenchyma and the characteristic unilateral pulmonary hyperlucency on radiographic images [9].

Diagnosis is usually made in childhood based on recurrent respiratory infections that encourage an active search for the cause, although in many other cases it goes unnoticed until adulthood, and the condition is often an incidental finding in asymptomatic patients [8,10]. The nonspecificity of its symptoms, including dyspnea, cough, hemoptysis, decreased exercise tolerance, and recurrent respiratory infections, makes it an underdiagnosed entity that leads to more frequent alternate and erroneous diagnoses such as COPD, asthma, pulmonary embolism, or pneumothorax [8,11]. Likewise, physical examination findings are nonspecific and include decreased thoracic expansion, decreased vesicular murmur with cramps or wheezing, and hyperresonance on percussion [12].

Imaging studies are key in the diagnosis of SJMS, especially chest X-ray, high-resolution (HRCT), CT angiography, MR angiography, or V/Q scan. The classic diagnostic triad includes (1) chest X-ray and HRCT with unilateral pulmonary hyperlucency associated with decreased ipsilateral pulmonary hilum caliber, and pulmonary artery with air trapping during expiration; (2) diffuse, decreased ventilation of the affected lung; and (3) decreased perfusion of the affected lung as evidenced by angiography or V/Q scan [3,5].

The main findings on a chest radiograph are unilateral hyperlucency with decreased bronchovascular markings and mediastinal shift to the affected side [4]. Chest CT complements the diagnosis by revealing hypoplasia or agenesis of the pulmonary artery, causing hypoperfusion of the pulmonary parenchyma with decreased vascularity and diffuse oligohemia [4], in addition, bronchiectasis or atelectasis may also be present; bronchiectasis is present in approximately 30% of patients and is directly related to the prognosis and intensity or frequency of clinical manifestations [13], since individuals with bronchiectasis present more severe exacerbations than those without this imaging finding [14].

Treatment is mainly conservative, based on respiratory therapy, optimization of bronchodilator management, pneumococcal and influenza vaccination, specific management of concomitant infections, and oxygen therapy in advanced cases [5,14]. Prevention, early identification, and treatment of recurrent respiratory infections are key in managing these patients [10]. Surgical management should be considered in patients with recurrent infections who do not have a satisfactory response to optimal medical management; surgical alternatives include pneumonectomy, lobectomy, or segmentectomy, and should be individualized for each case [14,15].

Yuce et al. published the first case of SJMS associated with severe pulmonary hypertension; this complication was diagnosed via right heart catheterization [16], and despite the uncertainty about its classification, the patient received bosentan and tadalafil, per the recommendations of the European Society of Cardiology (ESC). This led to a significant improvement in symptoms and functional class [16]. One hypothesis that could explain the association between SJMS and pulmonary hypertension is based on pulmonary arterial hypoplasia, which, combined with the loss of functional parenchyma and chronic hypoxic vasoconstriction secondary to poorly ventilated areas, generates a sustained increase in pulmonary vascular resistance; this progressive increase in pressure in the pulmonary circulation can lead to the development of pulmonary hypertension, even in the absence of diffuse parenchymal diseases [17]. These findings suggest that patients with SJMS and pulmonary hypertension may benefit from a targeted therapeutic approach with specific treatment for pulmonary hypertension, improving their prognosis and quality of life.

Limitations

This report has certain limitations, primarily the difficulty in definitively establishing the relative impact of each comorbidity on the development of pulmonary hypertension. The patient had a previous diagnosis of COPD, a condition that can also be associated with pulmonary hypertension, especially in advanced stages or with sustained hypoxemia. However, in this case, the characteristic imaging pattern, with left pulmonary artery hypoplasia, diffuse oligohemia, and localized bronchiectasis, as well as the favorable clinical and functional outcome following targeted treatment, support the hypothesis that SJMS was the primary pathophysiological mechanism involved.

## Conclusions

While SJMS is a rare and underdiagnosed entity, it is significant due to its severe complications and outcomes on the clinical condition and quality of life of patients. We discussed the case of an adult female with recurrent respiratory infections in childhood, in whom an incidental diagnosis of SJMS was made with pulmonary hypertension as a complication of the same. We believe this report will aid in current clinical practice by raising awareness of an underdiagnosed entity, such as SJMS, which is associated with serious complications and high impact on quality of life, such as pulmonary hypertension. Early identification and accurate diagnosis are essential for an adequate clinical approach in these patients. Treatment is mainly conservative and multidisciplinary, and, in a minority of cases, may require surgical management.
